# Midpoint Reflections on USAID HIV Local Partner Transition Efforts

**DOI:** 10.9745/GHSP-D-22-00338

**Published:** 2023-06-21

**Authors:** Deborah Kaliel, Christy Knight, Latham Avery, Lauren A. White, Lindsay Bonanno, Jason Porter, Kathryn Hoeflich, Courtney Irwin, Christopher Nikola, Amy Paul, Mai Hijazi, Clint Cavanaugh, E. Callie Raulfs-Wang

**Affiliations:** a Office of HIV/AIDS, Bureau for Global Health, U.S. Agency for International Development, Washington, DC, USA.; b Bureau for Management, Office of Acquisition and Assistance, U.S. Agency for International Development, Washington, DC, USA.; c Independent scholar, Washington, DC, USA.; d Office of Health Systems, Bureau for Global Health, U.S. Agency for International Development, Washington, DC, USA.

## Abstract

We discuss 5 key factors that have facilitated efforts to transition the majority of the USAID HIV/AIDS portfolio to direct funding through local organizations, including partner country governments.

## Key Messages

- Strengthening local systems through direct partnerships with local organizations and partner governments is a critical pathway to locally led development and achieving sustained epidemic control of HIV/AIDS.
- The U.S. Agency for International Development (USAID) has made substantial progress toward the U.S. President’s Emergency Plan for AIDS Relief goal of funding 70% of its portfolio directly through local organizations, including partner country governments.
- Five key factors that facilitated localization of the HIV/AIDS portfolio include: (1) developing strategic and intentional plans grounded in local reality; (2) customizing data systems to monitor progress toward goals and adapting them as needed; (3) investing in strengthening local partner organization and financial management capacity; (4) changing the way USAID does business; and (5) ensuring consistent USAID and local leadership and advocacy.
- Efforts in the first part of the HIV local partner transition have focused on reducing barriers on the USAID side; over the next 3 years, the HIV programs will need to focus on reducing barriers on the local partner side.

## INTRODUCTION

Historically, foreign assistance in global health has been characterized by a donor-driven paradigm whereby donor countries control the objectives and implementation of technical assistance programs.^1^ However, there has been an increasing focus in the global health community on the concept of country ownership, including in the context of HIV/AIDS.^1^^–^^4^ Although definitions of country ownership vary, this concept generally refers to partner countries and local organizations taking on increasing independence in designing, implementing, and financing their health programs.^2^ Country ownership is thought to support long-term sustainability of health care systems by increasing and maintaining scale of service delivery, improving efficiency of resource coordination, increasing integration with existing health care systems, and motivating domestic funding.^2^^,^^4^ Transition describes the process of moving from donor-led to local-led planning, managing, and delivery of health care programs.^4^^,^^5^ However, there is a lack of literature documenting many transition steps and how to best operationalize them. For example, a key intermediate step is when local organizations still receive U.S. Government (USG) funding and support but begin to operationalize programs themselves.^4^

In 2010, the U.S. Agency for International Development (USAID) announced the USAID Local Solutions goal of 30% of funding going directly to local entities by 2015. In 2016, some of the largest donors and humanitarian organizations signed the Grand Bargain, which included a goal of 25% of funding to local implementers by 2020.^6^ In November 2021, USAID Administrator Samantha Power outlined a “New Vision for Global Development” in a speech at Georgetown University, where she committed USAID to a 25% “local funding” target by 2025 and a 50% “local leadership” target by the end of the decade.^7^ Since the announcement of these goals, industry groups and development think tanks have published ubiquitous opinion pieces on “localization.”^8^^–^^10^

**FIGURE 1:**
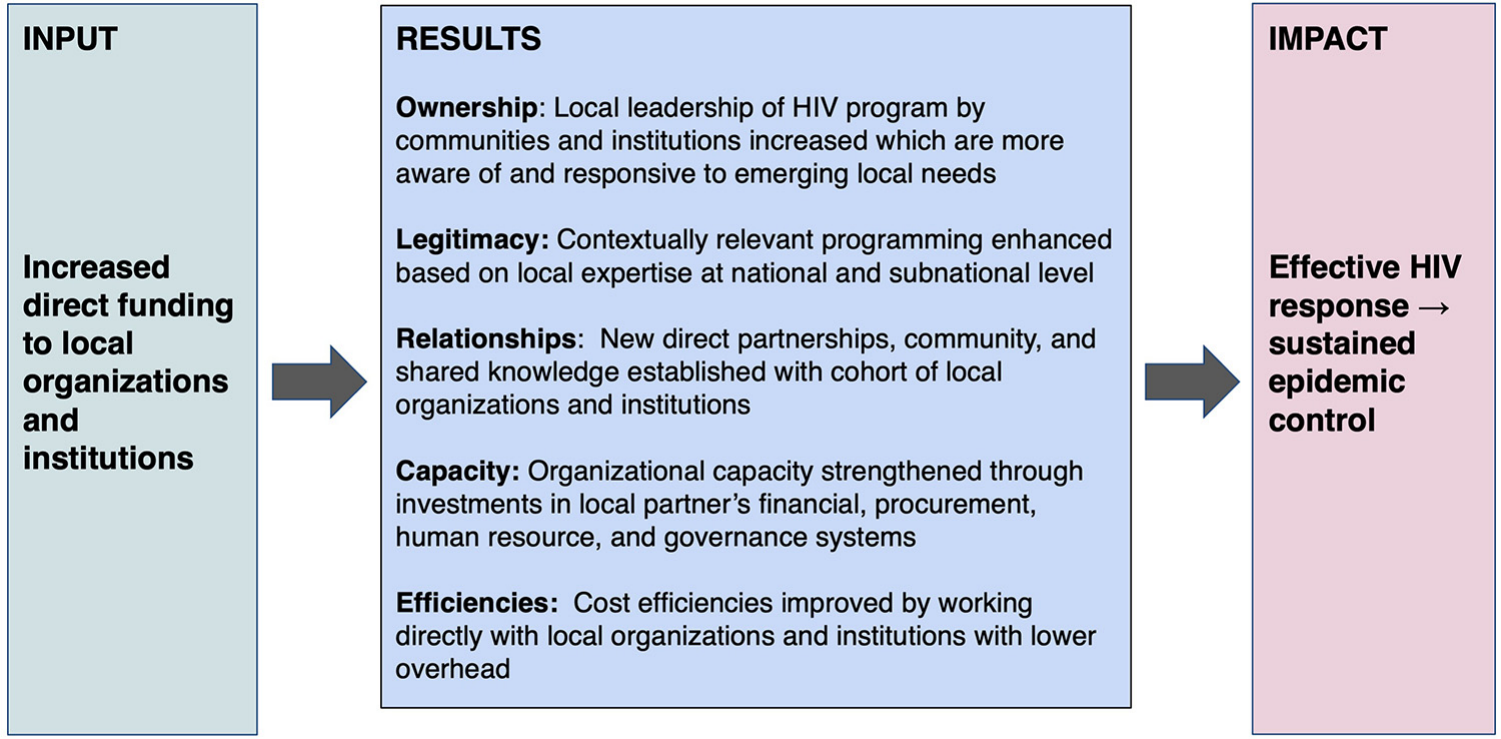
Direct Funding to Local Organizations for a Sustained HIV Response^a^ ^a^This conceptual framework describes how the local partner transition would improve HIV health outcomes and ultimately contribute to sustained epidemic control for HIV/AIDS. We highlight 5 interrelated potential results of increasing direct funding to local organizations and institutions that may support an effective HIV response: ownership, legitimacy, relationships, capacity, and increased efficiencies.

A key concept of HIV/AIDS program sustainability is the attainment and maintenance of sustained epidemic control whereby 95% of people living with HIV know their status, 95% of those tested are on treatment, and 95% of individuals on treatment are virally suppressed.^11^ We posit that direct funding of local organizations will support an effective HIV response and ultimately support sustained epidemic control in multiple, interrelated ways (Figure 1). Supporting local organizations directly means that communities and institutions that have a better understanding of local needs can provide responsive leadership and develop contextually relevant programming. Directly funding local partners creates a cohort of local organizations that foster community by developing new relationships, networking, and sharing knowledge. The prioritization of direct funding to local partners can create incentives to strengthen organizational capacity needed to elevate local actors in national and regional markets and diversify and strengthen the pool of local organizations able to successfully compete for and deliver global health programs. Finally, compared to traditional international partners that have historically delivered technical programming and assistance, working directly with local organizations can decrease expenditures for donor programs by reducing overhead costs.

Supporting local organizations directly means that communities and institutions that have a better understanding of local needs can provide responsive leadership and develop contextually relevant programming.

**FIGURE 2:**
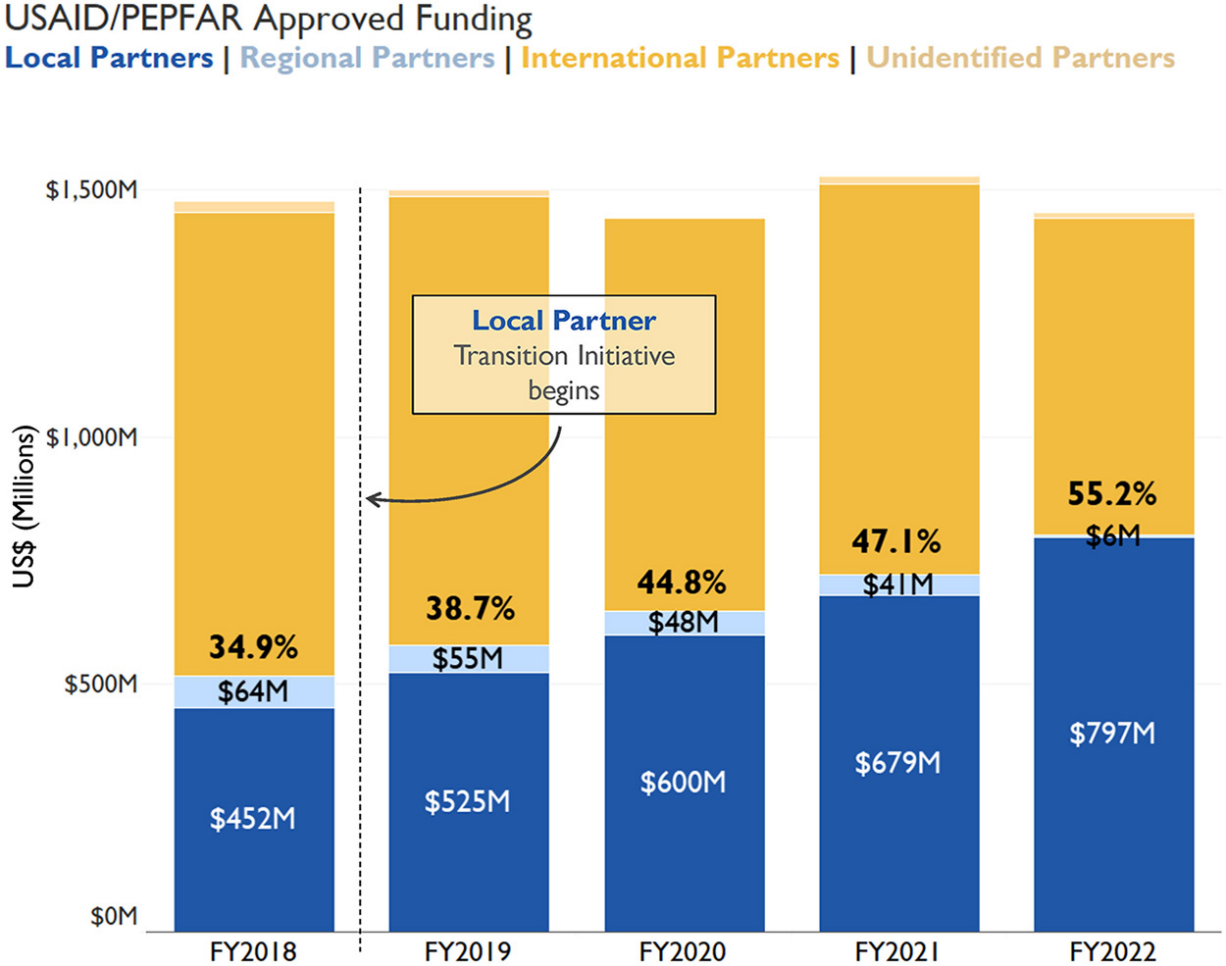
USAID/PEPFAR International vs. Local Partner Approved Funding Trends, FY2018–FY2022^a,b,c^ Abbreviations: FY, fiscal year; PEPFAR, U.S. President’s Emergency Fund for AIDS Relief; USAID, United States Agency for International Development; USD, United States dollar; USG, United States Government. ^a^The dashed line corresponds to the start of the PEPFAR local partner initiative in 2018. Aggregate global percent funding to local and regional partners was calculated by dividing the amount of USAID funding budgeted to local and regional partners by the total amount of funding budgeted to all partners. ^b^Local partners are incorporated in the country served by the PEPFAR program and either owned or staffed by a majority of citizens or legal residents of that country; regional partners are incorporated in another country in the region (as classified by the U.S. Department of State), rather than the specific country in which they are implementing. ^c^Calculations exclude USG management and operations costs, budgets for internationally procured commodities including the Global Health Supply Chain Procurement and Supply Management and Rapid Test Kits projects, and regional operating units. Funding data are derived from FACTS Info, an internal USG system (sourced March 31, 2023).

This commentary captures midpoint reflections from a USAID headquarters–based team supporting USAID HIV efforts to localize its portfolio in a relatively short amount of time. In 2018, the U.S. President’s Emergency Plan for AIDS Relief (PEPFAR) announced the goal of funding 70% of its program directly through local organizations, including partner country governments, by the end of 2020. At the inception, this was an incredibly ambitious goal given the 2-year timeline and that baseline levels of local partner funding for USAID’s HIV program were at just 34.9% in USG fiscal year (FY) 2018 (Figure 2). Additionally, it is worth noting that the 70% local funding was not announced as a special initiative or a stand-alone effort but as a clear expectation that teams would achieve in addition to meeting multiple other earmarks and targets required by the PEPFAR program.

**FIGURE 3:**
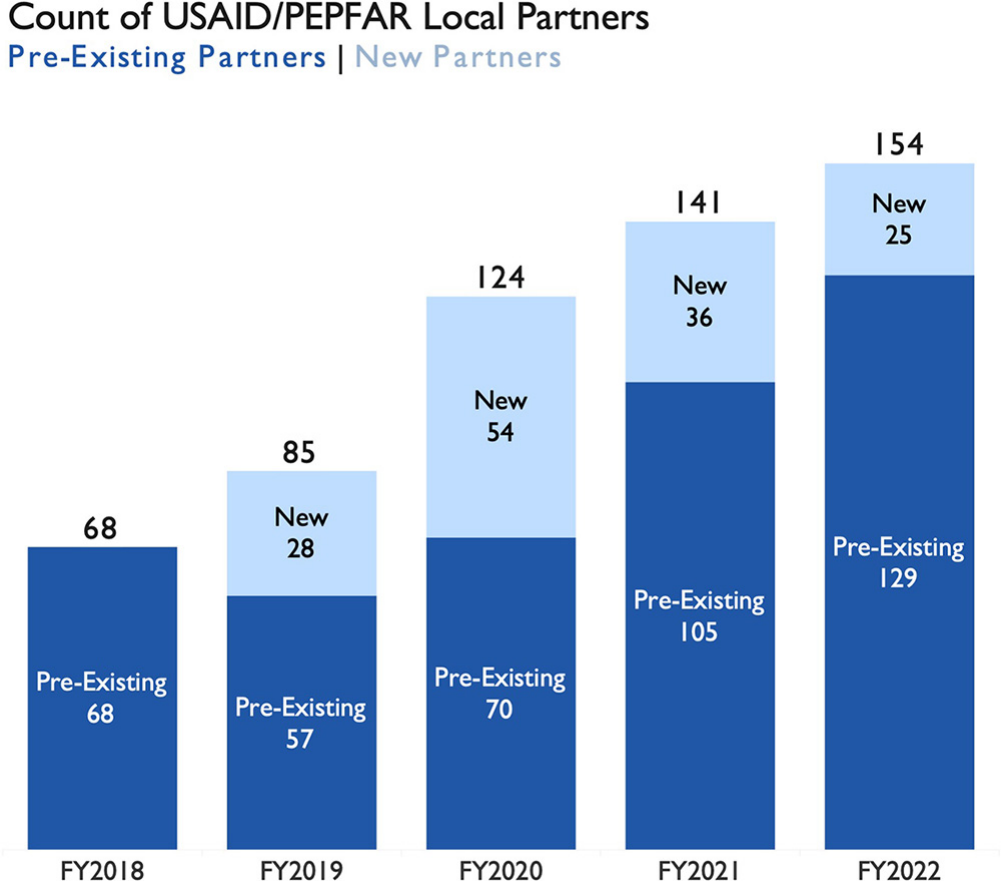
Composition of USAID/PEPFAR Preexisting and Newly Added Local Partners, FY2018–FY2022^a^ Abbreviations: FY, fiscal year; PEPFAR, U.S. President’s Emergency Fund for AIDS Relief; USAID, United States Agency for International Development. ^a^Bars represent a count of the number of local partners with direct USAID/PEPFAR awards each fiscal year. Preexisting partners are those that had a direct USAID/PEPFAR award before the Local Partner Transition (i.e., FY2018 or earlier) or those that have held a direct prime award for at least one year (e.g., a new partner in FY2019 is counted as a preexisting partner in FY2020). New partners each year are those who had not previously had direct awards with USAID/PEPFAR.

**FIGURE 4:**
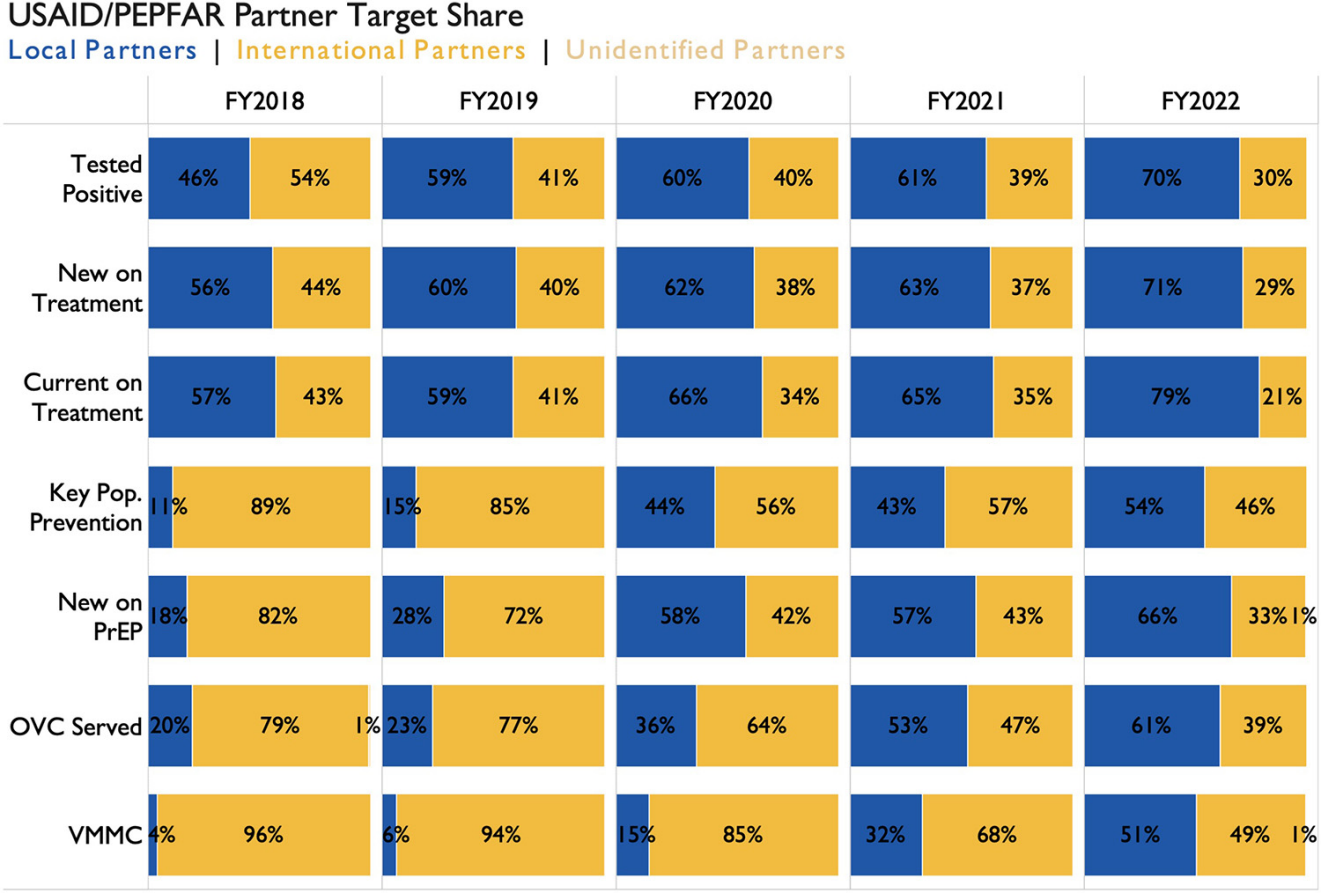
Global Aggregate Share of Approved MER Targets for USAID/PEPFAR International vs. Local and Regional Partners, FY2018–FY2022^a^ Abbreviations: FY, fiscal year; MER, monitoring, evaluation, and reporting; OVC, orphans and vulnerable children; PEPFAR, U.S. President’s Emergency Fund for AIDS Relief; PrEP, preexposure prophylaxis; USAID, United States Agency for International Development; VMMC, voluntary medical male circumcision. ^a^For each fiscal year, the proportion of USAID/PEPFAR targets for bilateral country programs is shown by partner type. Each row represents a separate MER indicator.

Since the 2018 announcement, USAID HIV programs have increased annual approved funding to local partners by US$345 million, or an 81% total increase since FY2018 (Figure 2). Currently, direct funding to local organizations represents 55% of USAID’s HIV program budget (Figure 2). At the country level, nearly every USAID HIV program has made progress in increasing annual funding and shifting programmatic performance targets to local organizations, and several USAID HIV programs have significantly localized their portfolios. USAID has brought on 143 new local partners since FY2018 (Figure 3). The majority of new partners are local nongovernmental organizations with a smaller number of local governments and private sector entities. Additionally, USAID HIV programs have shifted more than 50% of the responsibility for achievement against programmatic performance targets to local organizations (Figure 4). Major shifts in local implementation are particularly notable for programs focused on orphans and vulnerable children, preexposure prophylaxis, and key populations (Figure 4). The transition of both funding and programmatic targets to local partners occurred while USAID HIV programs continued to scale up service delivery and contributed to provision of lifesaving HIV treatment and quality services to nearly 4 million people.

Although PEPFAR is short of its 70% local partner funding goal, significant progress has been made in developing the capacity of local partners, including governments, to manage U.S. funding and strengthen local health networks in preparation for sustaining epidemic control efforts. Lessons on transitioning programs to local actors are also valuable to a larger audience of global health, development, and humanitarian aid practitioners because efforts to increase local funding are widely recognized as important but often difficult to achieve. We discuss 5 key factors that have enabled the significant shifts in funding, performance targets, implementation, and addition of new local partners in the USAID HIV localization agenda.

## 1. STRATEGIC AND INTENTIONAL PLANNING GROUNDED IN LOCAL REALITY

With the announcement of a 70% funding goal, a clear definition of which entities counted as “local” was published in the 2019 Country Operational Plan (COP) Guidance, which was easily referenced by both internal and external stakeholders.^12^ COP Guidance is drafted every year by the U.S. Department of State’s Office of the Global AIDS Coordinator that outlines annual PEPFAR programmatic priorities. To qualify as local, partners had to be locally incorporated, registered, and have a majority of local staff, including at the senior level. The definition was updated in the 2020 COP Guidance to include regional partners or those incorporated in the same geographic region as the country of implementation, although this only added marginally to the totals (Figure 2).^13^ The emphasis on the percentage of local staff in senior leadership positions and locally incorporated under the laws of the country of implementation made it clear that an international partner that established a locally affiliated office would not be considered local. The intent of the definition was to support organizations that had indigenous actors leading organizations. Subawards to local organizations were not included in the definition, which signaled PEPFAR’s commitment to prioritizing direct partnerships with local organizations.

In addition to a clear and public definition of a local partner, PEPFAR leadership communicated that the 70% funding goal was set for USAID HIV programs as a whole and not a requirement for each individual country, thus implying that countries had flexibility to transition programs to local implementation based on country-specific realities. Given that the 70% goal was global, it meant that some countries—especially those with larger overall budgets—would contribute more significantly to achieving 70% local funding.

Within 6 months of the local funding announcement, USAID leadership in the Office of HIV/AIDS convened an in-person meeting in South Africa for staff from the USAID missions and Washington, D.C., to support each country to develop their own local funding goals and strategies for FY2019–FY2021. To help inform the 3-year strategies, countries reviewed their FY2018 baseline data to better understand their current funding landscape and local partner funding opportunities for FY2019–FY2021. Following internal analysis of baseline data, country teams set funding goals based on local needs, context, and specific procurement opportunities. Strategies and funding goals were supported with detailed procurement planning documents that examined when current awards were ending and when opportunities for new awards were to be competed for locally.

Following internal analysis of baseline data, country teams set funding goals based on local needs, context, and specific procurement opportunities.

Concurrent with setting FY2019–FY2021 country-specific local funding goals, USAID country teams conducted a risk analysis to identify key risks associated with a substantial shift in funding to local partners. Commonly identified risks included: insufficient USAID staff and oversight, limited local partner capacity to manage direct awards, burdensome USAID policies and procedures, and poor internal and external communication and engagement. USAID country teams developed specific plans to manage and mitigate key risks in their FY2019–FY2021 transition plans. To foster transparency, collaboration, and sharing of best practices, country-specific local funding goals and associated risk analyses were disseminated across USAID country teams and HIV leadership. This process was recently repeated for the next 3 years of the PEPFAR local partner transition process, FY2022–FY2024.

Although the 70% timeline was ambitious, it created a sense of urgency needed to quickly mobilize a large bureaucracy. USAID country teams developed strategic approaches grounded in local reality and created concrete plans to help localize their portfolios. As a complement to the local funding projections, country-specific risk analysis helped country teams think through specific risks, vulnerabilities, and approaches to risk management. It is our opinion that without this ambitious goal and country-specific strategies resulting from it, USAID would have made far less progress.

Country-specific risk analysis helped country teams think through specific risks, vulnerabilities, and approaches to risk management.

## 2. CUSTOMIZING DATA SYSTEMS TO MONITOR PROGRESS AND ADAPTING THEM AS NEEDED

In addition to intentional and strategic planning around local partner transition, USAID and PEPFAR use several data systems to help the program monitor progress toward the 70% localization goal, monitor performance of new local partners, and course correct as needed.

Data systems to monitor partner performance against meeting programmatic performance targets (monitoring, evaluation, and reporting [MER]) and partner quality of service (Site Improvement through Monitoring System [SIMS]) already existed for all PEPFAR partners before the start of the local partner transition initiative. MER indicators describe performance against quantitative program targets for HIV/AIDS treatment, prevention, and support that are assigned to specific partners.^14^ SIMS data evaluate quality of services and programs at the facility, community, and above-site level.^15^ USAID closely tracks performance against MER targets and quality of services of local partners compared to international partners on a quarterly basis. Existing PEPFAR dashboards displaying SIMS or MER data were customized to visualize local partner-specific data. These data streams enable real-time tracking and monitoring of local partner performance relative to their international counterparts and assist USAID staff in triaging performance challenges and conducting necessary programmatic interventions.

To monitor percent local funding, PEPFAR also has a system of record (FACTS Info), which tracks approved COP funding by award and serves as the official record for annual local funding. FACTS Info is not a publicly accessible platform, but both budget and expenditure data are available at the partner level on Panorama Spotlight: https://data.pepfar.gov). FACTS Info data serve as a transparent and official record for local funding, but USAID needed a better way to monitor outyear funding projections, as well as annual progress toward funding goals. To meet this need, USAID developed an internal spreadsheet system that tracks planned USAID local awards for each PEPFAR country annually. Due to procurement sensitivities, individual country plans are access limited. Dollar values for planned local awards from each country are incorporated into a global aggregate tracked by USAID/Washington to determine progress against the global 70% target. This internal system helps identify and mitigate bottlenecks, for example, if a planned local award was delayed until the following year or needs more support from Washington through the procurement process. It also helps Washington staff clearly communicate to stakeholders at all levels about the status of the initiative.

## 3. INVESTING IN STRENGTHENING LOCAL PARTNER ORGANIZATION AND FINANCIAL MANAGEMENT CAPACITY

The country-specific risk exercise identified local organizational and financial capacity to manage USAID awards as a key risk in many countries. Additionally, USAID’s own capacity to manage local awards was identified as a key vulnerability for successful local partner transition efforts.

The country-specific risk exercise identified local organizational and financial capacity to manage USAID awards as a key risk in many countries.

As a response to the first risk, initial USAID efforts focused on landscaping existing subrecipients and preparing the most capable subrecipients for direct funding from USAID. Although USAID international partners had a deep roster of highly technical local subrecipients, many with more than a decade of experience implementing HIV programs, most of these subrecipients had little to no experience managing a direct award. USAID established several capacity-building mechanisms to rapidly assess and address gaps in local partner organizational and financial management capacity. Through these mechanisms, USAID ensured technical assistance was available for new local partners to help them navigate USAID prime partner requirements, including policy compliance and USG and PEPFAR reporting requirements. Organizational capacity-strengthening activities also included audit preparation; financial management and internal control processes; development of effective board and governance structures; and development of procurement, staffing, and human resource policies. The majority of these capacity-strengthening areas are critical for any organization’s successful operation, regardless of being a recipient of USG funding. In areas where missions could not easily or readily transition a program area to a local prime partner, mission staff focused on working with international technical assistance providers to strengthen the capacity of local partners who were issued subawards. Strengthening local partner organizational capacity helped reduce risks associated with the transition and facilitated the ability to shift funding to local organizations.

Strengthening local partner organizational capacity helped reduce risks associated with the transition to direct local funding.

To address concerns about USAID’s capacity to manage local awards, USAID missions conducted a staffing analysis to determine needed support over the next few years to manage an increase in local awards. Based on this analysis, USAID was approved to increase staffing levels by 98 positions across 16 missions. Mission teams hired staff in the USAID Health Office as well as the Offices of Acquisition and Assistance and Financial Management to help manage the entirety of local awards. Some missions also developed a “local transition” or “local capacity advisor” position explicitly focused on partner transition strategies and corresponding capacity support to local organizations. In Washington, USAID created a 10-person team dedicated solely to supporting local partner transition efforts. Staffing at Washington and missions focused on local partner award processes to help facilitate both the increased transition of awards to local primes, as well as ongoing program, financial, and quality monitoring, management, and support.

## 4. CHANGES IN THE WAY USAID DOES BUSINESS

USAID staff identified extended procurement timelines and internal processes as significant barriers to giving awards to local organizations and government partners. To reduce long procurement timelines, USAID relied on its HIV expedited procurement package and reduced internal procurement review timeline for HIV-related procurements to no more than 30 days by Agency leadership when the program requirement(s) was approved in the COP. USAID also increased funding thresholds for limiting competition for certain types of awards, which created additional opportunities for local partners ready to take on direct awards. For example, transition awards for USAID/PEPFAR programs were authorized for an increase from US$5 million to US$40 million. USAID also created more flexibility in the types of risk assessments that were required for direct government agreements. Although addressing these barriers reduced the timeline for new awards for USAID, they stopped short of reducing larger barriers of management and numerous reporting requirements for local organizations and government partners.

In addition to changing internal processes, USAID HIV programs have been intentional about increasing regular partner communication overall and building stronger relationships with new and existing local partners. To help inform and communicate country-specific funding goals, some USAID country teams developed vision statements that were shared with external stakeholders. Many teams also conducted landscape analyses to map potential local partners, held local stakeholder meetings to discuss local partner transition plans, or posted requests for information to help inform future procurements and solicit interest from local organizations.

Following the strategy-setting phase of USAID HIV local partner transition efforts, the Office of HIV/AIDS has regularly hosted implementing partner calls with both international and local partners to provide technical and programmatic updates to the partner community. Additionally, the Office of Acquisition and Assistance within the Bureau for Management prioritized partnership-building through quarterly business forecast calls with partner/trade representatives, including local organizations, and launched a “Work with USAID” website (https://www.workwithusaid.org/). At the country level, many missions hold monthly or quarterly implementing partner meetings with both international and local partners, which provide a forum to share best practices, coordinate programming, communicate technical directives, or share updates on the PEPFAR business processes. Several missions conduct joint site visits with implementing partners to health facilities or community-based groups, allowing for in-person programmatic observations and provision of immediate feedback.

In 2019, the Office of HIV/AIDS hosted the first annual Global Health Local Partner Meeting, a recurring forum for local partners to connect directly with each other and USAID staff to share technical and operational successes and innovations in HIV programming. The 2019 in-person meeting included 74 representatives from more than 40 local partners in 15 countries. The 2021 virtual meeting included representatives from 150 local partners (nongovernmental and government) and more than 1,000 virtual registrants. The 2022 hybrid meeting based in Johannesburg, South Africa, included 808 participants representing 189 local partners from 53 countries. At these annual fora, local partners have the opportunity to raise concerns and questions directly with USAID leadership and provide suggestions on how to improve partnerships. In addition, these meetings facilitate training and direct collaboration and networking between local partners in the absence of USAID.

The annual Global Health Local Partner Meeting facilitates training and the direct collaboration and networking between local partners in the absence of USAID.

## 5. CONSISTENT USAID AND LOCAL LEADERSHIP AND ADVOCACY

Strong leadership across multiple levels at USAID was critical for setting ambitious targets at the beginning and ensuring teams were equipped with tools and support to meet those goals. From the initial announcement at the level of the Global AIDS Coordinator through to USAID leadership across multiple bureaus and through to mission leadership, the message was consistent that the local partner transition was a priority because it would help to achieve programmatic aims. To support these USAID HIV local partner funding and strategic objectives as well as other cross-agency priorities, USAID created an oversight board—composed of senior-level representatives from relevant operating bureaus and offices (Office of the Chief Financial Officer, Bureau for Management Office of Acquisition and Assistance, Office of Human Capital and Talent Management, Bureau for Legislative and Public Affairs)—that reported to USAID overall leadership.

To facilitate leadership dialogue on USAID HIV local partner transition progress, the Office of HIV/AIDS hosted annual leadership calls with mission directors. These calls proved an opportunity for each country to share and review progress in their local partner transition objectives with both mission directors and the Office of HIV/AIDS leadership. They also provided a forum to raise cross-cutting issues.

The importance of the 70% local partner funding goal was woven into all priority documents and communications delivered by the Office of HIV/AIDS leadership, which facilitated many of the changes previously described, such as funding for capacity-building mechanisms, increased staffing, and reduction of procurement barriers. In several countries, the support from USAID mission directors proved pivotal, and missions with strong advocates served as models for other countries and were asked to share lessons across countries.

## CONCLUSIONS

Goals for increased direct funding to local partners have been set previously but were not fully operationalized or successful, often due to insufficient staffing and changes in or competing political priorities. Four years into the USAID/PEPFAR local partner transition efforts, it is important to reflect on what worked well for HIV programs and areas that could be improved.

Although the USAID HIV portfolio has not yet achieved the 70% funding goal, substantial progress has been made in strengthening local partner capacity and increasing direct funding and engagement with local partners over the last 4 years of intensive effort. The 70% goal was recently reaffirmed in COP 22 Guidance, although the specific time frame for achievement was removed. A recent USAID Office of Inspector General audit on the PEPFAR local partner transition, publicly released in December 2021, explored why USAID did not reach the funding goal by 2020 and concluded that “USAID’s PEPFAR budgets were not on track to meet local partner funding goals largely due to aggressive time frames driven by O/GAC [Office of the U.S. Global AIDS Coordinator and Health Diplomacy] and the low baselines that some missions started from.”^16^

Nevertheless, the 5 factors we describe supported a substantial shift in funding and programming to local organizations and government partners while still allowing for delivery of quality programs at scale. They also helped USAID HIV programs increase the number and diversity of direct local partners and could provide a model for similar transition efforts. While some of the particulars are specific to USAID and PEPFAR, we hope that the importance of these 5 factors has broader relevance for other localization efforts. The Box includes examples of questions to consider related to each factor.

BOX Factors and Questions to Consider When Localizing Program Funding

**1. Intentional planning:** Where are there tangible opportunities to increase local partnerships? What risks should be considered? Could they stand in the way of successful partnership? How can a concrete plan be established to reach a goal?

**2. Systems to monitor progress:** What are the baseline data (where is the organization starting from?)? What level of funding/partnership is the organization trying to reach? What data are necessary to track progress?

**3. Investment in capacity:** Where are the current capacity gaps? What capacities need to be prioritized to achieve the articulated goals?

**4. Changes in business processes:** What current business processes hamper the ability to make awards and partner with local organizations effectively?

**5. Consistent leadership:** Are leaders consistently communicating the importance of localization efforts and monitoring progress?

Efforts in the first part of the USAID’s HIV local partner transition focused on reducing barriers on the USAID side; over the next 3 years, USAID HIV programs will need to reduce barriers to make it easier for local partners to manage USG awards on the local partner side. Supporting these efforts will require a better understanding and elevating of local partners’ perspectives, as well as helping local organizations address long-term sustainability issues. USAID HIV programs will also need to tackle inequities in how USAID works with local partners versus international partners and ensure legal and contractual requirements are not unfairly advantaging international partners. Additionally, USAID will need to develop further skills and resources to work with smaller community organizations as well as government partners at the national and subnational levels. Strengthening local systems through direct partnerships with local organizations and partner governments is a critical pathway to locally led development and achieving sustained epidemic control of HIV/AIDS.

Over the next 3 years, USAID HIV programs will need to reduce barriers to make it easier for local partners to manage USG awards on the local partner side.

## Notes

**Peer Reviewed**

**First published online:** May 8, 2023.
